# Preliminary validation of bumby RAPE and MOLEST scales: Exploratory factorial analysis

**DOI:** 10.1080/07853890.2021.1896190

**Published:** 2021-09-28

**Authors:** Ricardo Ventura Baúto, Jorge Cardoso, Isabel Leal

**Affiliations:** a William James Center for Research – ISPA-IU, Lisboa, Portugal; b Centro de Investigação Interdisciplinar Egas Moniz (CiiEM), Egas Moniz Cooperativa de Ensino Superior, Caparica, Portugal; c Laboratório de Ciências Forenses e Psicológicas Egas Moniz (LCFPEM), Centro de Investigação Interdisciplinar Egas Moniz (CiiEM), Egas Moniz Cooperativa de Ensino Superior, Caparica, Portugal; d Laboratório de Psicologia Egas Moniz (LabPSI-EM), Centro de Investigação Interdisciplinar Egas Moniz (CiiEM), Egas Moniz Cooperativa de Ensino Superior, Caparica, Portugal; e Escola Superior de Saúde Egas Moniz (ESSEM), Caparica, Portugal

## Abstract

**Introduction:**

Cognitive distortions are considered an important element to understanding the support mechanisms of sexual aggression. Different Instruments have been developed for the assessment of sexual abuse and rape. In 1996, Bumby [1] developed two scales to assess cognitive distortions in these typologies of aggressors, MOLEST and RAPE. This paper aims to present the exploratory factor analysis of both instruments in a forensic sample and presents a proposal of factor structure with subscales, based on the conceptual models of Ward and Keenan [2] and Ward [3].

**Materials and methods:**

The data for this study were collected at Prison in Lisbon District (Portugal) between 2017-2019 after prior approval of the Directorate General for Reinsertion and Prison Services (DGRSP). From a total of 266 individuals (male) convicted by sexual abuse and rape, it was possible to obtain *N* = 71 (Age: *M* = 43.8 SD = 11.69; relations: 47.9% was married or in union; 32.4% has High School Education). Were excluded initially from sample, analphabets and non-Portuguese’s. All individuals were invited to participate in the study being explained that it was confidential and would not be benefitted or harmed for participate. The instruments used were MOLEST (38 items) and RAPE (36 items), both developed by Bumby (1996, Portuguese version by Baúto, Cardoso & Leal, 2014). For the data treatment was used IBM© SPSS© Version 20. All ethical principles have been considered and approved by the Portuguese Prison Services.

**Results:**

Significant levels of reliability and validity for both Scales were found. Regarding MOLEST Scale we tested the possibility of subscales, generated by grouping of items based on the varimax rotation method with principal component analysis. In the case of MOLEST, five sub-scales were defined and, in the RAPE, four sub-scales, categorised according to the Ward & Keenan [2] and Ward [3] models and defined as implicit theories. With reference to MOLEST we found a KMO = 0.607 (mediocre) with good Cronbach alphas for each of the five sub-scales (*α* = 0.912; *α* = 0.859; *α* = 0.856; *α* = 0.868; *α* = 0.799). These five subscales explain 44.85% of total variance. The RAPE has a KMO = 0.658 (mediocre) and four subscales have been defined which also have good Cronbach alphas (*α* = 0.867; *α* = 0.877; *α* = 0.839; *α* = 0.906). This sub-scale proposal explains 42.45% of the variance.

**Discussion and conclusions:**

The exploratory factorial analysis presents itself as promising, and it is necessary to use in the next phase a confirmatory factorial analysis that allows to test the consistency of the proposed model. The categorisation of the scale enhances its use in clinical practice, allowing the identification of primordial modes of functioning by the evaluated subjects.
